# Commentary: Mirror-Image Equivalence and Interhemispheric Mirror-Image Reversal

**DOI:** 10.3389/fnhum.2018.00375

**Published:** 2018-09-25

**Authors:** Jean-Paul Fischer, Christophe Luxembourger

**Affiliations:** Laboratoire Lorrain de Psychologie et Neurosciences, Université de Lorraine, Nancy, France

**Keywords:** mirror reversal, mirror equivalence, Corballis' theory, Orton's theory, character's orientation

In his recent theory of “mirror-image equivalence and interhemispheric mirror-image reversal,” Corballis (2018) suggests that confusion between an image and its mirror “is almost certainly a matter of recognition rather than perception *per se*.” (p. 3).

Additional evidence supporting Corballis' claim is provided by Fischer and Tazouti (2012, Expt. 1a), which compared the frequencies with which 5- to 6-year-old children reversed digits and letters when copying them and when writing them from memory (under dictation). They did this by asking one group of 143 children to copy the eight asymmetric digits and to write from memory eight asymmetric capital letters, and another group of 156 children to copy the same letters and to write from memory the same digits. The result was very clear, as the children reversed the characters much less frequently when they copied them (about 0.4% reversals) than when they wrote them from memory (more than 21% reversals). The rarity with which the children reversed the characters when copying them indicates that they perceive the left or right orientation of digits and capital letters almost perfectly.

Corballis made his claim when commenting on Rollenhagen and Olson (2000). In fact, Fischer and Koch (2016a, p. 121) made an analogous observation with respect to Blackburne et al. (2014), who wrote that “the remarkable ‘brain blindness’ to letter orientation in children is consistent with the view that letter perception begins developmentally with visual processes that are orientation insensitive” (p. 14). This view is not consistent with Corballis' theory or with the substantial difference in the frequency of reversal errors when copying compared with writing from memory.

Asymmetrical characters are perceived as “oriented” either toward the left (1, 2, 3, 7, 9, J, Z) or toward the right (4, 5, 6, B, C, D, E, F, G, K, L, N, P, Q, R, S), even by adults (Fischer, 2018). Children's reversals of left-oriented characters are generally due to them turning these characters in the direction of writing (left-to-right in Western cultures). That is, children who completely mirror-write (i.e., who write from right to left in Western cultures), reverse the right-oriented characters by turning them toward the left (Fischer, 2017).

Corballis' differentiation between the two hemispheres raises the question of how the reversed and veridical characters are distributed to them. Orton's (1925) well-known schema showing ABC in the left hemisphere and 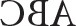 in the right hemisphere suggests a very simple categorization. However, Orton's hypothesis is difficult to accord with the knowledge of 4- to 5-year-old children, who cannot logically divide the characters into veridical vs. reversed characters for the simple reason that they do not know whether a character is veridical or reversed.

We have conducted many studies of reversals in consecutive writings of single characters by typically developing 5- to 6-year-old children (Fischer and Tazouti, 2012; Fischer, 2013, 2018; Fischer and Koch, 2016a,b). Combining results from these studies, we found, for example, reversal frequencies of 49.3% for the digit 3, vs. only 11.8% for the digit 4 (in writings by 1563 children), and reversal frequencies of 47.9% for the letter J, vs. only 4.6% for the letter K (in writings by 679 children). Figure 1 illustrates these reversals in the writings of one girl from our studies. Her reversals of 3, 1, 7, J, 9, Z, 2 are possible if the representations of the left-oriented characters are reversed in one hemisphere. This should allow the girl to produce her writings from memory by activating only the representations in this hemisphere. In contrast, Orton'shypothesis requires the girl to jump 15 times, for no obvious reason, from the representations in one hemisphere to the representations in the other hemisphere (see Figure 1). The girl's writings are also consistent with each hemisphere carrying both veridical and reversed representations, as in Corballis' theory.

**Figure 1:**
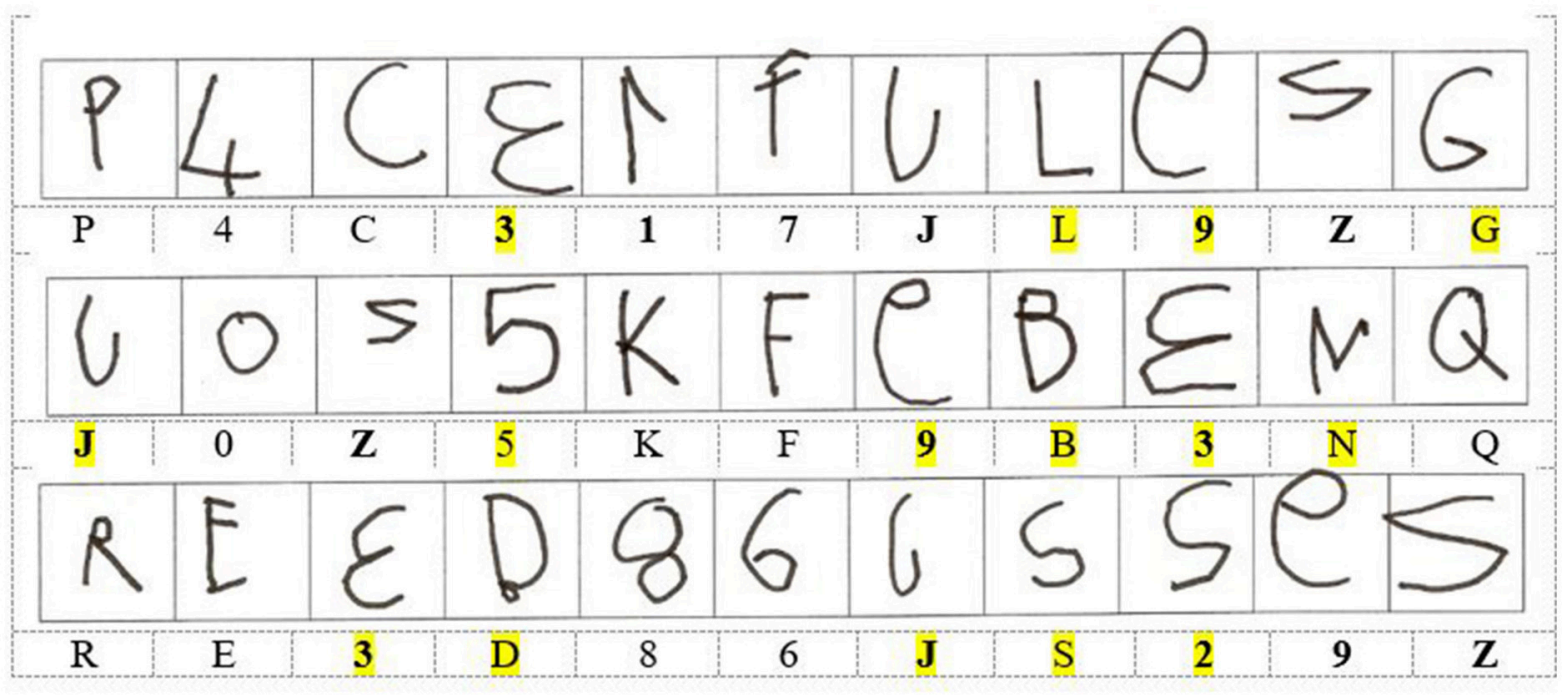
Writings of three series of independently dictated characters by a girl aged 6 years 2 months, who spontaneously wrote with her right hand (from raw data from Fischer and Tazouti, 2012, Expt 2). Mirror-reversed characters are shown in bold below the child's writings. Writings requiring a jump from a representation in one hemisphere to a representation in the other hemisphere, following Orton's (1925) hypothesis, are highlighted in yellow.

Orton's hypothesis comes up against the same problem in name writing. For example, HADJER, a boy aged 5 years 10 months (see Fischer, 2017) who mirror-wrote his name 
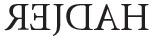
, would have had to recruit the representation of 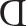 in the right hemisphere, the representation of J in the left hemisphere, and then return to the right hemisphere to write 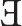 and 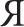. With veridical and reversed representations in each hemisphere, he could use representations of characters in only one hemisphere, thus avoiding any inexplicable jumps from one hemisphere to the other while writing his name.

A more precise hypothesis than the presence of veridical and reversed representations in both hemispheres is that one hemisphere contains reversed representations of the left-oriented asymmetrical characters and veridical representations of the other characters, whereas the other hemisphere contains reversed representations of the right-oriented asymmetrical characters and veridical representations of the left-oriented characters. This hypothesis would not only account for the girl's reversals presented in Figure 1, it would also explain another recent finding. In fact, children who use the representations in one hemisphere the most (because of its higher activation) should also be those who use the representations in the other hemisphere the least. Thus, the more precise hypothesis implies that children who reverse the left-oriented characters the most are also those who reverse the right-oriented characters the least. This has been shown to be the case for digits (Fischer, 2013), digits and capital letters (Fischer and Koch, 2016a), and capital letter-like characters (McIntosh et al., 2018a).

To conclude, Corballis' suggestion that “early processing retains left-right information for perception, but this is lost at the later stage where recognition takes place” (p. 4) is opportune. Furthermore, his theory's proposition that both hemispheres contain both veridical and reversed representations is compatible with character reversal in writing from memory as a function of a character's orientation, as observed by our research group and by Portex et al. (2018), McIntosh et al. (2018a,b), and Treiman et al. (2014). However, it does not exclude other explanations.

## Author contributions

J-PF was at the initiative of the paper, wrote the paper and was responsible of the publication process. CL has found financial support for the project, corrected and ameliorated the readability of the paper, and participated in all stages of the submission process.

### Conflict of interest statement

The authors declare that the research was conducted in the absence of any commercial or financial relationships that could be construed as a potential conflict of interest.
